# Factors Affecting Employees Use and Acceptance of Remote Working During the COVID-19 Pandemic: Evidence From the Jordanian Insurance Sector

**DOI:** 10.1177/21582440231181390

**Published:** 2023-06-22

**Authors:** Ibrahim N. Khatatbeh, Hashem Alshurafat, Mohannad Obeid Al Shbail, Fouad Jamaani

**Affiliations:** 1 Business School, The Hashemite University, Zarqa, Jordan; 2 Al Al-Bayt University, Mafraq, Jordan; 3 Taif University, Saudi Arabia

**Keywords:** remote work, COVID-19, Insurance industry, Social Capital Theory (SCT), Theory of Reasoned Action (TRA), technology acceptance model (TAM)

## Abstract

Remote working during the COVID-19 pandemic comes as an “enforced experiment,” where companies and individuals have turned to work from home to preserve business continuity. Drawing on a theoretical framework that integrates the Theory of Reasoned Action (TRA), Social Capital Theory (SCT), and Technology Acceptance Model (TAM), this research uses a sample of 134 survey responses to assess the factors affecting the acceptance and use of remote work during the COVID-19 pandemic among workers of the insurance industry in Jordan. The results suggest that social trust, perceived usefulness, and perceived ease of use can help elevate employee’s acceptance and use of remote work, whereas social norms have no significant effect. Considering these results, we further discuss implications and recommendations for the insurance sector.

## Introduction

Remote work, telework, virtual work, telecommuting, and e-work are synonyms to work done away from the “traditional workplace” (Sullivan, 2003). Vyas and Butakhieo (2021, p. 64) defined remote work within the COVID-19 context as “ an alternative working to minimize the risk of COVID-19 infection.” Conventionally, remote work is driven by many rationales, such as flexibility, advances in information and communication technology, and environmental concerns (reduce carbon emissions) (Green et al., 2020; Hilbrecht et al., 2013; Mello, 2007; Sia et al., 2004). Now, health safety was on top of these drivers due to the outbreak of the COVID-19 pandemic. The importance of remote work was spotlighted following the disruption of economic activity and mobility. Hence, the prevalence of remote work during the pandemic was instantaneous.

The unfortunate event of the COVID-19 pandemic has provided the opportunity for the largest work-from-home experiment ever happened (Bloomberg, 2020); it has been called “the Great Lockdown,” referring to the severe containment measures enforced due to the pandemic outbreak (Khatatbeh et al., 2020). Governments worldwide have taken extreme measures, such as lockdowns, to control the fast-spread COVID-19 pandemic. Therefore, companies and individuals have participated in an involuntary working-from-home experience to sustain business despite the disruption caused by the pandemic.

Jordan was no exception; the first confirmed COVID-19 case in Jordan was announced on March 2nd, 2020. The total number of confirmed cases has surpassed 50 by March 18th. Subsequently, the Jordanian authorities ruled out a strictly forced complete lockdown, which comes into effect on March 18th, to fight the spread of the pandemic. Therefore, it is crucial to understand that the remote working situation was not voluntary for workers in the Jordanian insurance sector. In fact, it was suddenly forced by the complete lockdown (curfew) in the early days of the pandemic spread in Jordan.

The available literature on remote working does not provide a thorough investigation of “government-enforced” remote working situations (Waizenegger et al., 2020). In fact, existing research has been conducted under normal business circumstances with no forces to work from home situation, as is the case under the threat of the COVID-19 pandemic, which gives no clue for factors affecting remote work in times of crises such as the current pandemic (Green et al., 2020). Sun Life (2020) stresses that both companies and individuals have seen the pandemic circumstances as an opportunity to test the remote working system. In this context, the desire to remote work has shown quite a jump since the start of the pandemic. According to a survey conducted by JLL (2020), which involved over 2,000 workers across 10 different countries, the acceptance of remote working by employees has doubled compared to the pre-pandemic period. The survey found that the average employee is now willing to work remotely 2.4 days a week, which is an increase from the 1.2 days per week reported in earlier surveys conducted prior to the pandemic. Moreover, the survey reports that more than 75% of workers prefer working remotely on a regular basis (JLL, 2020).

The Jordanian insurance sector was selected as the focus of this study for several reasons. First, the insurance industry is an important sector in the Jordanian economy, with a significant number of employees working in this sector (AL-Rjoub, 2018). Second, the insurance sector is highly relevant to remote working, as it involves a combination of client-facing and office-based work (Musgrave-Burdette, 2002). This presents unique challenges in terms of how employees adapt to remote work, and how organizations manage remote teams while maintaining a high level of client service. Additionally, the insurance sector has specific regulatory requirements and standards that may be affected by the shift to remote work (Abramovsky & Kochenburger, 2016). Therefore, the Jordanian insurance sector was an appropriate and relevant choice for this study on remote working during the COVID-19 pandemic.

This paper aims to assess the factors affecting employees’ acceptance and use of remote working in the Jordanian insurance sector during the COVID-19 pandemic. To achieve this objective, we surveyed employees in the Jordanian insurance sector. Our analysis of the survey data and interviews revealed several key findings. The results suggest that social trust, perceived usefulness, and perceived ease of use can help elevate employees’ acceptance and use of remote work, whereas social norms have no significant effect. The results of this study are of utmost importance for companies and their stakeholders in the Jordanian insurance sector, as well as in closely related sectors, by improving the effectiveness of remote working during and beyond the COVID-19 pandemic. The remainder of this paper is organised as follows. The next section provides an overview of the Jordanian insurance industry. Section three reviews the relevant prior studies. Section four illustrates the research model and develops the research hypotheses. Section five illustrates the research methodology. The research results are presented in section six and discussed in section seven. The final section concludes the paper.

## An Overview of the Jordanian Insurance Industry

The origins of the insurance industry can be dated back to the 1940s. AL-Rjoub (2019) provides a comprehensive account of the development of the insurance sector in Jordan. Currently, 24 insurance companies are operating in Jordan; which comprises 15 companies that offer non-life, life, and medical insurance, 7 companies offer non-life and medical insurance, a life insurance company, and a non-life insurance company (JIF, 2019). The share of total written premiums of the insurance sector to GDP was extremely stable in recent years, hovering around 2%, from 2015 to 2019.

In 2020, the consolidated gross premiums of operating insurance companies were equal to JOD 594 million, accounting for a 3.5% decrease from 2019. Non-life insurance lines (Motor, Marine, Aviation, Fire, Liability, Medical, etc..) dominate the business with more than 84% (JOD 501.7 million) of the total gross premiums. Motor insurance and medical insurance are the major non-life insurance lines ad account for 36% and 30% of the gross insurance premiums, respectively. The remaining 16% of the total gross premiums are generated from the life insurance business (JOD 92.3 million). On the other side, surprisingly, the gross paid insurance claims plummeted to JOD 4.11.1 million in 2020, down from JOD 490.3 million in 2019 (about a 16.1% decline). The gross paid non-life insurance claims decreased to JOD 375.4 million in 2020, about a 13.9% decline from 2019. Similarly, gross paid life insurance claims reported a 34% fall to reach JOD 35.7 million. Reported complaints have also declined by 35% to reach 655 complaints in 2020; most of these complaints, about 97%, come from motor insurance. The substantial decline in claims and complaints may be attributed to the decline in economic activity and mobility due to the lengthy lockdown and strict curfew measures forced by the government to fight the spread COVID-19 pandemic.

## Literature Review

The employee’s acceptance and use of remote work is an issue that has been examined with respect to many sectors, prior to the COVID-19 pandemic. Historically, the rates of planned remote work as an employment option were usually low (Galanti et al., 2021; Green et al., 2020; Milasi et al., 2021). However, the event of the COVID-19 health crisis provides realistic evidence, although under extreme circumstances, that remote work could have a larger share as an employment option. For instance, Fana et al. (2020) provide a study case where remote work accounts only for 4% and 2.5% in 2019 in Spain and Italy, respectively. These statistics went over tenfold during the pandemic period, as of April 2020, to reach 40% in Italy and 30% in Spain.

Accordingly, the successful adoption of remote work varies considerably across sectors, and it is highly pertinent to white-collar occupations (high-skilled, high-paid, top-, and middle-management occupations) and information- and technology-intensive sectors (Taser et al., 2022; Fana et al., 2020; Milasi et al., 2020). Mckinsey, the famous global management consulting firm, provides a progressive analysis of remote work for 2,000 tasks in 800 jobs and nine countries. They find that the overall potential of remote work is highly relevant to tasks and activities carried out within different occupations. Accordingly, the finance and insurance sectors have the biggest potential to apply for remote work without loss of productivity, particularly, 76% to 86% of time spent on activities that can be done remotely (Lund et al., 2020).

The successful implementation of remote work in the insurance sector represents both an opportunity and a challenge to insurance companies. Gigi and Sangeetha (2020) find that employee performance and satisfaction are highly leveraged by the effectiveness of communication among employees. Green et al. (2017) argue that companies may avoid remote working options due to limited control of communication and management. Therefore, manager’s openness to explore and support alternative working options and develop employee’s perception of trust and autonomy are highly significant factors (Green et al., 2020; Kröll et al., 2017). Moreover, Waizenegger et al. (2020) show that remote worker’s productivity is largely impacted by the three classes of affordance: technological, environmental, and social affordances. For instance, technological affordance was “actualised as supporting functional affordances to complete tasks,” and facilities a wide range of collaboration among employees, such as virtual teams and conference meetings.

### Benefits and Challenges of Working Remotely

The reviewed literature demonstrates both benefits and challenges of working remotely, associated with individuals and organizations. To begin with, improved worker’s performance (productivity) is the top-cited in the literature (Gajendran & Harrison, 2007). This may be explained by the following findings: First, employees who work remotely experience fewer disruptions through the course of their tasks (Bailey & Kurland, 2002). Second, remote workers have more time available by saving commuting time (Apgar, 1998; Howe et al., 2022). Third, remote workers can adjust their working time and environment to achieve the most productive setup (Baltes et al., 1999; Gajendran & Harrison, 2007; Kłopotek, 2017). Finally, remote workers are less likely to take short absences due to illness (Anderson & Kelliher, 2020). Remote working is also associated with higher job satisfaction, retention, and better work-life balance (Gajendran & Harrison, 2007). Martin and MacDonnell (2012) list the benefits of remote working at the organizational level, notably, increased worker’s productivity, retention, turnover intent, commitment, and performance.

On the contrary, some workers may face overwork issues, where the intensity of working remotely more than 2 or 3 days per week is associated with increased family-work conflict (Bentley et al., 2016, Gajendran & Harrison, 2007; Golden et al., 2008; Green et al., 2020). Moreover, remote workers may encounter social isolation, which negatively impacts their performance (Green et al., 2020). Vyas and Butakhieo (2021) conducted a SWOT analysis for work from home and work from office options. Whereas, Kłopotek (2017) discovered that younger workers value face-to-face communication in the workplace and therefore may experience decreased productivity when working remotely.

Green et al. (2020) argue that the major challenge in remote working is to maintain the employee’s well-being and performance in the event of social isolation forced by the pandemic. Moreover, this challenge is affected by other challenges that would limit the practicality of remote work. Particularly, they identify three other key challenges that are directly related to well-being and performance outcomes, these are: (i) the availability of sufficient technology with proper training and support, (ii) limited communication and management (interactions of co-workers and management), and (iii) the lack of a dedicated physical home working environment that is comparable to the traditional workplace environment. In the same vein, Verbeemen and D’Amico (2020) suggest three main areas of remote work that companies need to address for a successful implementation in the long term: (i) Implementing remote working in a structural way, (ii) Securing the infrastructure for remote working, (iii) Balancing the work and private lives of employees.

### The Future of Remote Work

Thanks to COVID-19, remote work has become the “new normal” during the pandemic and may continue afterwards. Therefore, nearly all business sectors (including the insurance sector) must adapt to this changing environment and develop tools to reap opportunities and tackle associated challenges. In this sense, the International Labor Organization (ILO) has issued “An employers’ guide on working from home in response to the outbreak of COVID-19,” which stresses the importance of working remotely as the COVID-19 crisis end is “not yet in sight.” Moreover, only one-third of CEOs surveyed in the KPMG CEO outlook survey (March 2021) expect the return of regular business sometime in 2021, compared to 45% who anticipated the return of regular business in 2022 (KPMG, 2021).

The views toward remote work as a permanent option are changing quickly. On one hand, the pandemic has brought an enforced experiment of remote work, which was handled successfully, and most companies managed to enhance their remote working arrangements. Green et al. (2020) argue that more employers and employees would rather the remote working situation continue either fully or partially when business returns to normal. Likewise, Lund et al. (2020) suggest that hybrid models of conventional-remote work that emerged during the lockdown periods are likely to continue post-pandemic. Remarkably, over 60% of this research participants have conveyed their preference for an option to work remotely in some capacity in the post-lockdown period.

On the other hand, enthusiasm toward remote working is regressing, compared to the beginning of the pandemic. The KPMG CEO Outlook survey, which includes insights from 500 CEOs across 11 key markets (Australia, Canada, China, France, Germany, India, Italy, Japan, Spain, the United Kingdom, and the United States), reveals that executives are now reconsidering their stance on office space reduction and are instead focusing on developing strategies to engage their employees in planned remote working systems. Specifically, a recent KPMG CEO Outlook survey, March 2021, shows that only 30% of the CEOs plan to engage employees in planned working from home for 2 to 3 days a week. Moreover, the percentage of CEOs considering reducing office spaces has declined to 17% from the 69% reported in the August 2020 survey.

Nevertheless, one may argue that the experience of working remotely during the pandemic is still in its infancy and has not matured enough to be adopted as a working option when businesses return as usual. However, this enforced experiment provides an opportunity to weigh the benefits and costs of remote work as an alternative employment option (whether full or partial), and examine the employee’s acceptance and use of remote working option for business success and continuity.

## Research Model and Hypotheses Development

**Figure 1.**
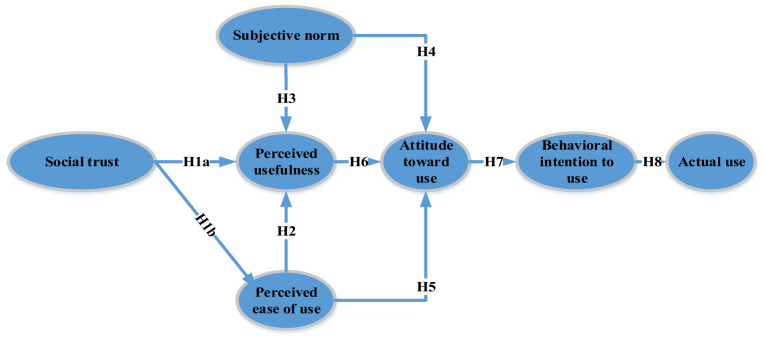
The research theoretical framework (Ajzen & Madden, 1986; Alshurafat, Al Shbail, Masadeh, et al., 2021; Davis, 1989).

This research integrates TRA, SCT, and TAM to explore the factors that affect remote working within the Jordanian insurance industry. Despite the broad utilization of TRA, SCT, and TAM in prior studies (Ajzen & Madden, 1986; Al Shbail et al., 2021; Awadallah & Elgharbawy, 2021; Bourdieu, 1985; Davis et al., 1989; Dumpit & Fernandez, 2017; Fishbein & Ajzen, 1977; Lee et al., 2003; Montaño & Kasprzyk, 2015; Putnam et al., 1993), few, if any, have integrated TRA, SCT, and TAM to predict and explain insurance sector employees’ acceptance and use of remote working in developing countries. Figure 1 shows the integrated theoretical framework.

TRA is a social psychological theory that explains how attitudes and norms influence behavior (Alshurafat, Al Shbail, & Almuiet, 2021; Awadallah & Elgharbawy, 2021; Fishbein & Ajzen, 1977; Raza & Khan, 2021; Raza et al., 2021). According to TRA, individuals are more likely to perform a behavior if they have a positive attitude toward it and perceive that important others expect them to perform it (Hale et al., 2003; Montaño & Kasprzyk, 2015). SCT is a sociological theory that explains how social networks and relationships can create value for individuals and organizations (Jaradat et al., 2022; Munoz-Leiva et al., 2017; Qureshi et al., 2021; Raza, Umer, et al., 2020). According to SCT, social capital is created through relationships and networks that provide access to resources, information, and support (Dumpit & Fernandez, 2017). SCT has been used in studies of organizational behavior and technology adoption, as it provides a framework for understanding the role of social networks and relationships in facilitating or hindering technology adoption and use (Compeau et al., 1999; Schunk, 2012).

TAM is a psychological theory that explains how individuals form perceptions of technology and make decisions about using it (Mailizar et al., 2021; To & Trinh, 2021). According to TAM, individuals are more likely to use technology if they perceive it to be useful and easy to use (Ali et al., 2020; Alshurafat et al., 2023; Daragmeh et al., 2021; Raza, Qazi, Shah, et al., 2020; Raza, Qazi, Umer, & Khan, 2020; Sani et al., 2020). TAM has been widely used in studies of technology adoption and use, as it provides a framework for understanding how individuals form perceptions of technology and how these perceptions influence their behavior (Al Shbail et al., 2022; Ashrafi et al., 2021; J. Liu et al., 2019).

These three theories were integrated in this study to provide a more comprehensive understanding of the factors that influence employees’ acceptance and use of remote working in the Jordanian insurance sector during the COVID-19 pandemic. By using TRA, SCT, and TAM together, we were able to identify the key factors that influence remote working acceptance and use and provide a more nuanced understanding of how these factors interact.

Many theorists and researchers have recognized social trust as a fundamental factor influencing individuals’ perceptions of the expected efforts and benefits of certain behavior (Bourdieu, 1985; Coleman, 1994; Nahapiet & Ghoshal, 1998; Putnam, 1992; Putnam et al., 1993; Woolcock, 1998). Misztal (1996) defines trust as the belief that the “results of somebody’s intended action will be appropriate from our point of view.” In this study, the theoretical model suggests that the level of social trust impacts the perceived ease of use and the perceived usefulness (Dumpit & Fernandez, 2017; Venkatesh et al., 2003).

In the TAM model, the perceived ease of use and perceived usefulness are essential factors affecting the attitude toward using new technologies (Muchran & Ahmar, 2019; Munoz-Leiva et al., 2017; Soneka & Phiri, 2019). Davis (1989, p. 320) has defined perceived usefulness as “the degree to which a person believes that using a particular system would be free of effort.” This follows from the definition of “ease”: “freedom from difficulty or great effort.” Perceived ease of use genuinely comprises psychological beliefs of users’ perceptions toward the easiness of performing tasks using new technologies (Alhashmi et al., 2019; Gangwar et al., 2015; Pedrosa et al., 2020). Davis (1989, p. 320) has defined perceived usefulness as “the degree to which a person believes that using a particular system would enhance his or her job performance.”Grant et al. (2013) and Slavković et al. (2022) reported that trust is a significant factor that positively impacts remote working effectiveness. Based on these fundamentals, the following hypotheses have been formulated:

*Hypothesis 1A: High social trust leads to high perceived usefulness.*
*Hypothesis 1B: High social trust leads to high perceived ease of use.*

The influence of perceived ease of use on the attitude to adopt and use new technologies has been examined in many prior studies (Zhou et al., 2021). For instance, Park and Kim (2014) found that perceived ease of use is an influential factor affecting attitudes toward the use of mobile cloud services. In another study, Rafique et al. (2020) reported that the perceived ease of use is an influential factor that positively impacts users’ attitudes to adopt mobile library applications. Therefore, the following hypothesis has been formulated:

*Hypothesis 2: perceived ease of use positively impacts the perceived usefulness.*

As proposed by Fishbein and Ajzen (1977), who invented the theory of reasoned action (TRA), in certain circumstances, subjective norms are factors that restrict and influence someone’s attitude toward behavior (Awadallah & Elgharbawy, 2021). Referring to the TRA, the principal purpose of the TRA is to understand people’s intentional behavior by observing the fundamental motivation to act. Besides, the normative construct of the theory (i.e., social norms encircling the behavior) also contributes to whether people will adopt the behavior (Ajzen & Madden, 1986; Fishbein & Ajzen, 1977). Connor and Sparks (2005, p. 174) define subjective norm as “a function of normative beliefs, which represent perceptions of specific significant others’ preferences about whether one should or should not engage in a behavior.”Wang et al. (2021) reported that the subjective norm is a significant factor that positively impacts the perceived usefulness of remote working. Therefore, the following hypothesis has been formulated:

*Hypothesis 3: Subjective norms positively impact the perceived usefulness.*

The attitude toward a use behavior is expected to be influenced by the factors mentioned earlier (perceived usefulness, perceived ease of use, and subjective norms). The influence of subjective norms on the attitude toward a behavior has been examined in many prior studies. For example, Raza et al. (2019) found that subjective norm has a significant impact on consumers’ attitude toward Islamic insurance. Similarly, as hypothesized by the TAM model, I.-F. Liu et al. (2010) found that the attitude to adopt and use technology is significantly affected by the perceived ease of use and perceived usefulness. Thus, the following hypotheses have been formulated:

*Hypothesis 4: Subjective norms positively impact the attitude toward a use.*
*Hypothesis 5: Perceived ease of use positively impacts the attitude toward a use.*
*Hypothesis 6: Perceived usefulness positively impacts the attitude toward a use.*

The TRA asserts that the attitude toward using a certain behavior precedes the behavioral intention to use (Ajzen & Madden, 1986; Alshurafat, Al Shbail, Masadeh, et al., 2021; Fishbein & Ajzen, 1977; Hale et al., 2003; Montaño & Kasprzyk, 2015; Nisson & Earl, 2020). On the other hand, the TAM model predicts the impact of behavioral intention on the actual behavior (Davis et al., 1989; Lee et al., 2003; I.-F. Liu et al., 2010; Marangunić & Granić, 2015). Based on these fundamentals, the following hypotheses have been formulated:

*Hypothesis 7: High attitude toward use leads to behavioral intention to use*
*Hypothesis 8: High behavioral intention to use leads to actual usage.*

## Research Methodology

### Sample Description

Employees from Jordanian insurance firms comprised the study’s sample. The first author gathered the data. The survey was created first in English and then translated into Arabic. Three experts independently translated the survey back into English to ensure no information distortion during translation. Microsoft forms were used to collect the data. Participants in the study participated willingly and were not incentivized to do so. They were chosen using the snowball sampling method since the majority of related research uses the same method. A toal of 147 employees returned the questionnaires, but only 134 were considered complete and valid for analysis. The study’s sample size parameters were consistent with the literature (Comrey & Lee, 1992). A pilot study was conducted prior to the actual study to validate the reliability of the questionnaire.

Table 1 shows the respondents’ demographic profile. Out of 134 respondents, 94 (70.1%) are males and 40 (29.9%) are females. Also, 110 (82.1%) respondents have a bachelor’s degree. About 101 (75.4%) respondents are working in insurance companies. Moreover, 43 (32.1%) of the respondents have more than 15 years of work experience in the business, 35 (26.1%) of the respondents have 10 to 15 years of work experience, 28 (20.9%) of the respondents have 5 to 10 years of work experience, 28 (20.9%) of the respondents have below 5 years work experience.

**Table 1.** Respondents’ Profile.

Demographic variables	Category	Frequency	(%)
Gender	Male	94	70.1
	Female	40	29.9
Academic achievement	Bachelor	110	82.0
	Master	12	9.0
	Other	12	9.0
Specialization	Risk management and insurance	33	24.6
	Finance	8	6.0
	Accounting	6	4.5
	Business administration	28	20.9
	Economics	8	6.0
	Other	51	38.0
Work nature	Insurance companies	101	75.4
	Reinsurance companies	7	5.2
	Insurance brokerage companies	11	8.2
	Medical insurance management companies	9	6.7
	Other	6	4.5
Experience	<5 years	28	20.9
	>5 <10 years	28	20.9
	>10 <15 years	35	26.1
	>15 years	43	32.1

### Measurements of Variables

The instrument was constructed to include a four-part questionnaire, with the first part consisting of nominal scales and the remainder consisting of 7-point Likert scales. As a consequence, the first section includes general information. This section of the questionnaire was used to gather basic information about the employees’ characteristics, such as gender and educational level. The questionnaire’s second segment is used to assess PU and PEOU in the TAM adapted from Davis (1989) and Venkatesh and Davis (1996, 2000) studies. Four items to measure intention to use the system based on Lin (2011) and two measures of actual system use are separately assessed based on Hubona and Kennick (1996) and Moon and Kim (2001) studies. The number of hours per week that staff registered using the system is used to determine utilization frequency. The number of hours per week that staff registered using the system is used to quantify the consumption rate. The third section of the questionnaire is focused on attitude and SN, which are adapted from prior studies by Ajzen (1991), Pavlou (2003), and Taylor and Todd (1995); all have five items. SCT is discussed in the fourth section of the questionnaire. Three items based on an instrument developed by Mathwick et al. (2008) were used to evaluate social trust.

### Common Method Variance Test

The research ensured that the prevalent method bias did not occur before running the measurement model. This evaluation affirms that the variances are reflected in the calculation process rather than the construct itself. Harman’s (1976) one-factor test and Podsakoff’s (2003) guidance were used to achieve this. All items on the measurement scale were subjected to a principal component analysis with the varimax rotation in order to identify any signs of a single factor from the factor analysis. The first factor accounted for 33.50% of the variance, according to the results. This value is consistent with the general rule of less than 50%. (Podsakoff et al., 2003).

## Results

### Measurement Model

**Figure 2.**
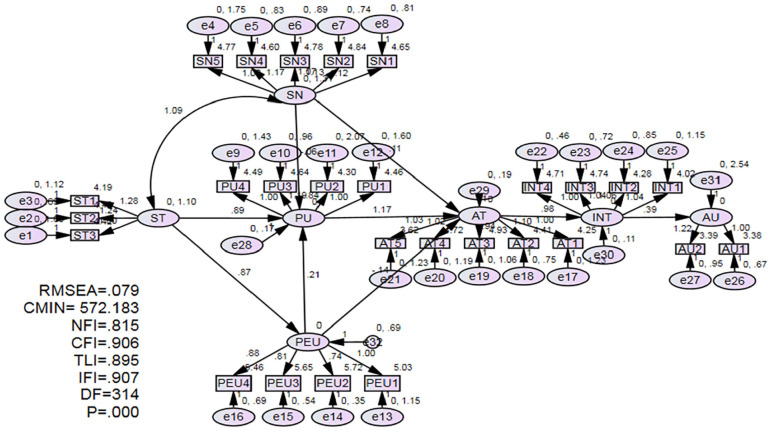
Structural equation model.

AMOS 25.0 was used to assess the model’s psychometric capabilities using confirmatory factor analysis (CFA) and calculate the proposed theoretical model using SEM. There were no missing data points, and the variables were usually distributed. Each factor was measured and improved using CFA (Arbuckle, 2010). CFA models must have an appropriate fit based on the following criteria: root mean square error of approximation (RMSEA) 0.08; Tacker–Lewis index (TLI) >0.90; comparative fit index (CFI) >0.90; and incremental fit index (IFI) >0.90 (Hooper et al., 2008). In this study, RMSEA = 0.079, *df* = 314, NFI = 0.815, CFI = 0.906, TLI = 0.895, and IFI = 0.907, indicating acceptable model fit (see Table 3 and Figure 2).

Cronbach’s alpha values for all variables observed; values greater than .8 suggest high internal consistency (Al-Olimat & Al Shbail, 2020; Sijtsma, 2009). The measurement model’s convergent validity was checked using factor loadings and average variance extracted (AVE). Factor loadings (ranging from 0.71 to 0.95) were significant (*p* < .001) and dropped between the 0.50 and 0.95 suggested threshold values (see Table 2). The AVE values ranged from .618 to .906, which surpassed the .50 threshold for high convergent validity (Fornell & Larcker, 1981). According to the findings, the build items accounted for more than half of the variance for each latent factor. Furthermore, the build reliability surpassed the .5 threshold value (see Table 2). All thresholds were met, suggesting that the items for each research variable were internally consistent and accurate (Al-Olimat & Al Shbail, 2020; Al Shbail et al., 2018; Eldalabeeh et al., 2021). As a result, all latent variables were fairly reliable.

**Table 2.** Measurement Scales and Reliability.

Construct	Code	*M*	*SD*	CFA standardized loading	C-α	CR	AVE
Social trust	ST-1	4.187	1.707	0.879	.772	.868	.689
	ST-2	4.455	1.539	0.875			
	ST-3	4.418	1.72	0.727			
Subjective norms	SN-1	4.649	1.571	0.857	.888	.918	.691
	SN-2	4.843	1.554	0.874			
	SN-3	4.784	1.547	0.838			
	SN-4	4.604	1.616	0.865			
	SN-5	4.769	1.749	0.713			
Perceived usefulness	PU-1	4.455	1.709	0.792	.794	.865	.618
	PU-2	4.299	1.728	0.710			
	PU-3	4.642	1.681	0.865			
	PU-4	4.493	1.656	0.768			
Perceived ease of use	PEU-1	5.03	1.634	0.834	.873	.913	.724
	PEU-2	5.716	1.09	0.866			
	PEU-3	5.649	1.235	0.845			
	PEU-4	5.455	1.369	0.858			
Attitude toward use	AT-1	4.254	1.773	0.839	.903	.928	.722
	AT-2	4.41	1.75	0.908			
	AT-3	4.925	1.664	0.819			
	AT-4	4.724	1.789	0.836			
	AT-5	3.619	1.811	0.843			
Intention to use	INT-1	4.022	1.797	0.863	.916	.941	.798
	INT-2	4.284	1.735	0.906			
	INT-3	4.739	1.675	0.902			
	INT-4	4.709	1.549	0.902			
Actual usage	AU-1	3.381	1.872	0.954	.896	.951	.906
	AU-2	3.388	2.269	0.950			

### Structural Model

The structural model was tested after the measurement model was determined to be accurate and satisfactory. The nomological relationships were explored in the second stage using SEM (Al-Naimat et al., 2020; Al Shbail et al., 2018) among the seven latent constructs: (a) direct relationships between ST and both PU and PEU, (b) direct relationship between PEU and PU, (c) direct relationship between SN and PU, (d) direct relationships between SN and AT, (e) direct relationships between PEU and AT, (f) direct relationships between PU and AT, (h) direct relationships between AT and INT, and (h) direct relationships between INT and AU (see Figure 2).

The structural model was tested using significance tests for estimated coefficients, which provided evidence to support or deny the proposed relationships between the construct variables (Al-Olimat & Al Shbail, 2020; Al Shbail et al., 2018; Shbail & Shbail, 2020). The following metrics were chosen to assess how well both the measurement and structural models match the data: RMSEA, df, NFI, CFI, TLI, IFI, and *p*. Table 3 shows that RMSEA = 0.079, *df* = 314, NFI = 0.815, CFI = 0.906, TLI = 0.895, and IFI = 0.907. The fit indicator analysis showed that the measurement model and structural model offered an acceptable fit. According to the model evaluation results (see Table 3), the seven latent variables in the measurement model demonstrated high reliability and validity, and the model had a good fit (see Table 3).

**Table 3.** Model Fit For Structural Equation Model.

Model	Result	Criteria
RMSEA	0.079	≤0.080
*df*	314	
NFI	0.815	≥0.90
CFI	0.906	≥0.90
TLI	0.895	≥0.90
IFI	0.907	≥0.90
*p*	0.000	*p* > .05

The hypotheses were tested using the structural model. There were 27 observed items and seven latent variables in the measurement model (see Table 2). To investigate the eight hypotheses, the proposed structural model (see Figure 2) defined the following relationships: ST would have an impact on PU and PEU; PEU would have an impact on PU; SN would have an impact on PU; PU would have an impact on AT; SN would have an impact on AT; PEU would have an impact on AT; AT would have an impact on INT, and INT would have an impact on AU. Six of the hypotheses were found to be supported, except for the influence of SN on PU (β = −.061, *t* = −0.254), the effect of SN on AT (β = .110, *t* = 0.646 < 1.96), and the effect of PEU on AT (β = −.143, *t* = −1.061 < 1.96). In structural analysis, *t*-value metrics coupled with critical ratios surpassed the critical value (1.96) at the level *p* < .01(Al-Olimat & Al Shbail, 2020; Eldalabeeh et al., 2021; Obeid et al., 2017).

The standardized effects of ST had a positive influence on PU (β = .886, *t* = 2.745) and PEU (β = .867, *t* = 5.873). The results show that when ST was increased by 1 *SD*, PU increased by 0.886 standard deviations, and PEU increased by 0.900 standard deviations. Therefore, H1a and H1b were supported. PEU have a positive influence on PU (β = .208, *t* = 2.007). Therefore, H2 was supported. SN did not have a positive influence on PU (β = −.061, *t* = −0.254 < 1.96). The standardized effects of SN did not influence AT (β = .110, *t* = 0.646 < 1.96). Therefore, H3 and H4 were not supported. PEU did not have a positive influence on AT (β = −.143, *t* = −1.061 < 1.96). Therefore, H5 was not supported. PU was a positive influence on AT (β = 1.174, *t* = 4.342). The results show that when PU was increased by 1 *SD*, AT increased by 1.174 *SD*. Therefore, H6 was supported. The standardized effects of AT positively influence INT (β = .976, *t* = 11.448). The results show that when AT was increased by 1 *SD*, INT increased by 0.976 standard deviations. Therefore, H7 was supported. The standardized effects of INT positively influence AU (β = .389, *t* = 3.358). The results show that when INT was increased by 1 *SD*, AU increased by 0.389 *SD*. Therefore, H8 was supported (Table 4).

**Table 4.** SEM Results for Structural Model.

Hypothesis	Relationship			β	*T*	*p*	Decision
H1a	ST	→	PU	.886	2.745	.006	Supported
H1b	ST	→	PEU	.867	5.873	***	Supported
H2	PEU	→	PU	.208	2.007	.045	Supported
H3	SN	→	PU	−.061	−0.254	.799	Not supported
H4	SN	→	AT	.110	0.646	.518	Not supported
H5	PEU	→	AT	−.143	−1.061	.289	Not supported
H6	PU	→	AT	1.174	4.342	***	Supported
H7	AT	→	INT	.976	11.448	***	Supported
H8	INT	→	AU	.389	3.358	***	Supported

*** *p* < .001.

## Discussion

Remote working systems are an indispensable solution for the insurance sector in different contexts during the COVID-19 period. This study examined factors influencing the adoption and acceptance of remote working within the Jordanian insurance sector using an integrated theoretical framework that encompasses TRA, SCT, and TAM (Ajzen & Madden, 1986; Awadallah & Elgharbawy, 2021; Bourdieu, 1985; Davis, 1989; Davis et al., 1989; Dumpit & Fernandez, 2017; Fishbein & Ajzen, 1977; Lee et al., 2003; I.-F. Liu et al., 2010; Putnam, 1992; Woolcock, 1998). This has been achieved by gathering the perceptions of the employees who are working in the Jordanian insurance companies through a previously tested and valid questionnaire. Consequently, based on this research’s findings, it is evident that different factors influence the employees’ acceptance of remote working. However, these factors show different levels of influence on remote working.

Based on the findings, social trust is an influential factor that impacts both the perceived usefulness and perceived ease of use of remote working within Jordanian insurance companies. These results are consistent with previous research on technology adoption and use (Almaiah et al., 2020; Dumpit & Fernandez, 2017; Venkatesh et al., 2003). These results highlight the importance of trust in the decision toward working remotely. Therefore, companies within this research context are encouraged to maintain and promote the level of trust among employees who are working remotely. This could be achieved by spreading and supporting new technological culture among the employees, allowing them to trust and accept technological changes without fear of later negative sequences.

Similarly, the perceived ease of use of remote working has been considered an influential factor that impacts the perception of insurance companies’ employees regarding the perceived usefulness of adopting and using remote working systems. This means that the employees think that the use of remote working systems will benefit them as much as it is easy to be used to complete their tasks. This result is aligned with other research in the field (Davis, 1989; Davis et al., 1989; Dumpit & Fernandez, 2017; Lee et al., 2003; I.-F. Liu et al., 2010). Consequently, insurance companies’ managers are requested to inform their employees about the easy ways of completing their job tasks using remote working systems. This can be done by providing training courses for the employees on how to use remote working systems.

The research findings show some interesting and unexpected results concerning the impact of social norms on the perceived usefulness of remote working systems and the attitude toward to use of remote working systems. In this research, the hypotheses were built based on the common trend of the aggregate empirical results from the theory and previous studies (Ajzen & Madden, 1986; Awadallah & Elgharbawy, 2021; Fishbein & Ajzen, 1977; Hale et al., 2003; Montaño & Kasprzyk, 2015; Venkatesh et al., 2003). However, the results refute the hypotheses concerning social norms. A possible justification for these results is that the employees are forced by the pandemic conditions to work remotely as all businesses worldwide have been overwhelmed by the massive impact of the COVID-19 pandemic. Therefore, an employee who is compelled to use remote working systems would find the impact of others meaningless when it comes to deciding whether to adopt and use remote working systems or not. Another interesting result is related to the impact of the perceived ease of use on the attitude to the use of remote working systems. The same justification is valid here. In detail, the easiness degree of using remote working systems would not shape the decision of an employee who is compelled to use remote working systems.

This research indicates that the attitude toward use decision as a dependent variable is well predicted by one major predictor which is the perceived usefulness. Thus, it can be reasoned that the perceived usefulness factor is the most significant factor that can make the adoption and transformation to a remote working system a successful process. Furthermore, the attitude toward use is an influential factor that affects behavioral intention to use, while the latter positively affects the actual usage. These results are similar to what has been found by previous researchers on technology adoption and use (Almaiah et al., 2020; Davis, 1989; Davis et al., 1989; Lee et al., 2003; I.-F. Liu et al., 2010; Marangunić & Granić, 2015; Raza et al., 2019; Venkatesh et al., 2003; Wang et al., 2021). Therefore, the implication of these findings suggests that employees would show more acceptance of the remote working systems that provide them with the maximum benefit in their work.

### Theoretical and Practical Implications

The findings of this study have several theoretical and practical implications. From a theoretical perspective, our study contributes to the existing body of knowledge by integrating three theoretical frameworks: The theory of Reasoned Action (TRA), Social Capital Theory (SCT), and the Technology Acceptance Model (TAM). Using this integrated framework, this paper identified the key factors influencing employees’ acceptance and use of remote working in the Jordanian insurance sector during the COVID-19 pandemic. This study has contributed to the theoretical understanding of remote working acceptance and use in the Jordanian insurance sector during the COVID-19 pandemic and has practical implications for organizations in this sector and beyond. By building on our findings, researchers can continue refining our understanding of the factors influencing remote working acceptance and use.

From a practical perspective, our findings have important implications for organizations in the Jordanian insurance sector and related sectors. Our study highlights that social trust, perceived usefulness, and perceived ease of use can help elevate employees’ acceptance and use of remote work, whereas social norms have no significant effect. By addressing these factors, organizations can improve employees’ acceptance and use of remote work, increasing productivity, and well-being.

### Future Research Recommendations

This study provides valuable insights into the factors that affect employees’ acceptance and use of remote working during the COVID-19 pandemic in the Jordanian insurance sector. However, several areas warrant further investigation. First, our study focused on the Jordanian insurance sector, and it would be interesting to examine whether this study’s findings generalize to other sectors and countries. Second, this study relied on self-reported data from employees, and future research could benefit from additional objective measures. Third, our study focused on the COVID-19 pandemic, and it would be helpful to examine the factors that influence remote working in non-pandemic contexts. Finally, our study focused on the perspectives of employees and managers, and future research could benefit from additional perspectives, such as those of clients or customers.

## Concluding Remarks

This study aimed to assess the factors affecting the acceptance and use of remote work in the Jordanian insurance sector during the COVID-19 pandemic. To achieve this, a survey was created and distributed to employees in the insurance sector in Jordan, and 134 survey responses were collected and analyzed. The results confirm that remote work is an essential option during the pandemic, and factors such as social trust, perceived usefulness, and perceived ease of use influence employee acceptance and use of remote work. However, social norms do not significantly impact employee decisions to work remotely, likely due to the pandemic’s forced circumstances. These findings have significant implications for the Jordanian insurance sector and other related industries, as remote work may become a flexible employment option.

The interest in remote work has been revived due to the necessity forced by the COVID-19 pandemic. The findings of this study provide useful insights for insurance companies and other industries considering adopting remote work beyond the pandemic period. Remote work can become a fully or partially flexible option of employment. To fully adopt remote work, companies should define metrics and develop new measures for productivity and performance, including reliable communication tools and a reward system for employee self-discipline and loyalty. Furthermore, building trust with remote employees and monitoring their performance is crucial for effective remote work. Overall, this study sheds light on the challenges and opportunities associated with remote work and emphasizes the need for companies to embrace it beyond the pandemic period.
